# Respiratory Viruses in Hospitalized Children with Influenza-Like Illness during the H1n1 2009 Pandemic in Sweden

**DOI:** 10.1371/journal.pone.0051491

**Published:** 2012-12-14

**Authors:** Samuel Rhedin, Johan Hamrin, Pontus Naucler, Rutger Bennet, Maria Rotzén-Östlund, Anna Färnert, Margareta Eriksson

**Affiliations:** 1 Department of Medicine Solna - Infectious Diseases Unit, Karolinska University Hospital, Stockholm, Sweden; 2 Department for Women and Child Health, Karolinska University Hospital, Stockholm, Sweden; 3 Astrid Lindgren Children’s Hospital, Stockholm, Sweden; 4 Department of Microbiology, Tumor and Cell Biology, Karolinska Institutet, Stockholm, Sweden; 5 Department of Infectious Diseases, Karolinska University Hospital, Stockholm, Sweden; 6 Department of Clinical Microbiology, Karolinska University Hospital, Stockholm, Sweden; University of Hong Kong, Hong Kong

## Abstract

**Background:**

The swine-origin influenza A(H1N1)pdm09 pandemic of 2009 had a slower spread in Europe than expected. The human rhinovirus (HRV) has been suggested to have delayed the pandemic through viral interference. The importance of co-infections over time during the pandemic and in terms of severity of the disease needs to be assessed.

**Objective:**

The aim of this study was to investigate respiratory viruses and specifically the presence of co-infections with influenza A(H1N1)pdm09 (H1N1) in hospitalized children during the H1N1 pandemic. A secondary aim was to investigate if co-infections were associated with severity of disease.

**Methods:**

A retrospective study was performed on 502 children with influenza-like illness admitted to inpatient care at a pediatric hospital in Stockholm, Sweden during the 6 months spanning the H1N1 pandemic in 2009. Respiratory samples were analyzed for a panel of 16 viruses by real-time polymerase chain reaction.

**Results:**

One or more viruses were detected in 61.6% of the samples. Of these, 85.4% were single infections and 14.6% co-infections (2–4 viruses). The number of co-infections increased throughout the study period. H1N1 was found in 83 (16.5%) children and of these 12 (14.5%) were co-infections. HRV and H1N1 circulated to a large extent at the same time and 6.0% of the H1N1-positive children were also positive for HRV. There was no correlation between co-infections and severity of disease in children with H1N1.

**Conclusions:**

Viral co-infections were relatively common in H1N1 infected hospitalized children and need to be considered when estimating morbidity attributed to H1N1. Population-based longitudinal studies with repeated sampling are needed to improve the understanding of the importance of co-infections and viral interference.

## Introduction

Influenza pandemics have struck human societies throughout history, sometimes with devastating outcome [1]. In March 2009, the novel swine-origin influenza A(H1N1)pdm09 (referred throughout as H1N1) was reported in Mexico causing great fear in countries worldwide [2], [3]. The spread in Europe was however slower than expected [4]. In Sweden, the first sporadic cases were diagnosed in May 2009 and not until October, after a smaller peak in July and August, the reported cases increased to peak in November 2009 [5]. Sweden was one of the countries that adopted countrywide vaccination against H1N1 reaching a coverage of approximately 60% of the population, with the first doses distributed in the middle of October 2009, and vaccination may have contributed to the rapid decline in reported cases in December [6].

The delayed spread of H1N1 in Sweden during the early autumn of 2009 has been suggested to be due to viral interference with human rhinovirus (HRV) [7]. Also in Norway and France, high PCR detection ratio of HRV in early autumn before the spread of H1N1 was reported [8], [9], but the hypothesis of viral interference between H1N1 and HRV has been contradicted in studies from Italy and USA [10], [11]. Viral interference refers to that infections with one virus affect the spread of other viruses reflecting either an epidemiological or a biological interaction, which in turn could be due to a cellular or immunological mechanism [7], [12], [13]. Although the H1N1 pandemic has been extensively investigated there are limited studies that have assessed viral co-infections over time and their importance in severity of disease during the pandemic.

We investigated viral real-time polymerase chain reaction (PCR) data of respiratory samples and clinical records in hospitalized children with influenza-like illness (ILI) in Stockholm, Sweden during the H1N1 pandemic in 2009. The aim of this study was to investigate respiratory viruses and specifically the presence of co-infections with influenza A(H1N1)pdm09 (H1N1) in hospitalized children during the H1N1 pandemic. A secondary aim was to investigate if co-infections were associated with severity of disease.

**Figure 1:**
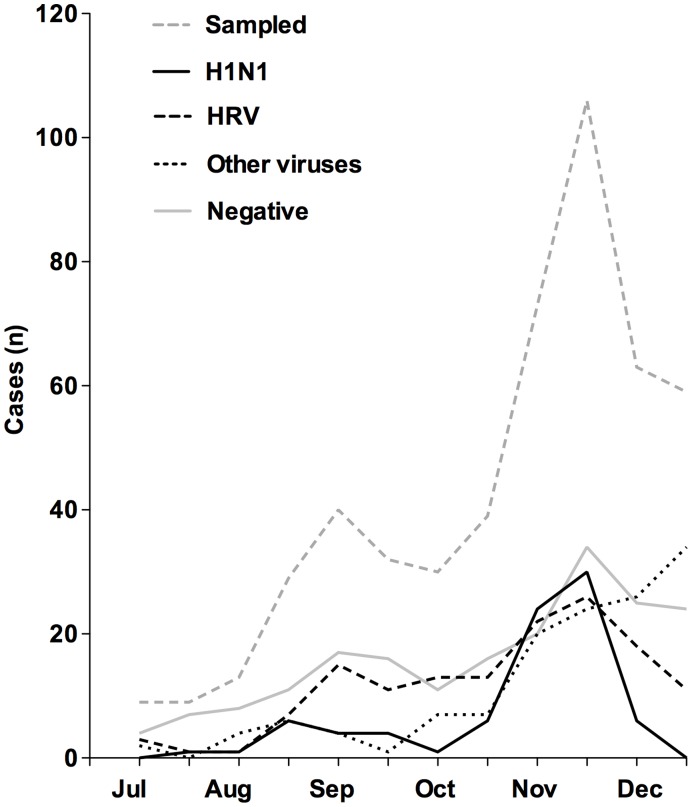
Prevalence over time. Total number of detected viruses among children with influenza-like illness at Astrid Lindgren Children’s Hospital, Karolinska University Hospital, Stockholm during the influenza A(H1N1)pdm09 pandemic. Included is also the number of PCR-negative children (neg, n = 193) as well as total number of sampled children (n = 502).

**Table 1:** Viral co-detection among PCR-positive samples.

Detected virus, n (%)												
	H1N1	HAdV	HBoV	HCoV	HEV	HMPV		HRV	RSV	PIV1	PIV2	PIV3
Positive samples^a^	83 (16.5)	29 (5.8)	29 (5.8)	17 (3.4)	13 (2.6)	2 (0.4)	141 (28.1)		12 (2.4)	22 (4.4)	9 (1.8)	2 (0.4)
Single infections	71 (85.5)	18 (62.1)	11 (37.9)	6 (35.3)	12 (92.3)	1 (50.0)	111 (78.7)		11 (91.7)	15 (68.2)	8 (88.9)	1 (50.0)
Co-infections^b^	12 (14.5)	11 (37.9)	18 (62.1)	11 (64.7)	1 (7.7)	1 (50.0)	30 (21.3)		1 (8.3)	7 (31.8)	1 (11.1)	1 (50.0)
H1N1	–	3 (10.3)	4 (13.8)	1 (5.9)		1 (50.0)	5 (3.5)			1 (4.5)		
HAdV	3 (3.6)	–		1 (5.9)			8 (5.7)			1 (4.5)		
HBoV	4 (4.8)		–	3 (17.6)	1 (7.7)	1 (50.0)	9 (6.4)			1 (4.5)	1 (11.1)	
HCoV	1 (1.2)	1 (3.4)	3 (10.3)	–			6 (4.3)			1 (4.5)		1 (50.0)
HEV			1 (3.4)		–							
HMPV	1 (1.2)		1 (3.4)			–	1 (0.7)					
HRV	5 (6.0)	8 (27.6)	9 (31.0)	6 (35.3)		1 (50.0)	–		1 (8.3)	3 (13.6)	1 (11.1)	
RSV							1 (0.7)		–			
PIV1	1 (1.2)	1 (3.4)	1 (3.4)	1 (5.9)			3 (2.1)			–		
PIV2			1 (3.4)				1 (0.7)				–	
PIV3				1 (5.9)								–

a Number in brackets representing percentage of total children (n = 502).

b Triple and quadruple infections counted as multiple double infections.

## Materials and Methods

### Study Design

The study was a retrospective study of children 0–17 years with suspected influenza admitted to inpatient care at Astrid Lindgren Children’s Hospital at Karolinska University Hospital in Solna, a tertiary university hospital in Stockholm, Sweden between July 1 and December 30 2009, the period of the H1N1 pandemic in Stockholm. The catchment area covered 221 585 children 0–17 years [14]. The policy of the hospital during the H1N1 pandemic was to collect respiratory samples for viral PCR analyses from every child who was admitted to inpatient care with clinical suspicion of influenza i.e. ILI, including children with unspecific symptoms, such as abdominal pains, vomiting, diarrhea, seizures and apnea (in infants).

Children admitted to inpatient care in which a respiratory sample was analyzed by viral PCR during the study period were identified at the accredited (ISO 15189∶2007) Microbiological Laboratory at Karolinska University Hospital. The children were excluded if the respiratory samples had initially been tested solely for H1N1 and respiratory syncytial virus (RSV) and the material was insufficient for analysis of additional viruses or if the respiratory samples were taken without clinical suspicion of influenza i.e. surveillance testing when children were moved to the pediatric intensive care unit or elective bronchoscopy. If a child was sampled more than once, only the first sample was included in the analyses.

**Figure 2:**
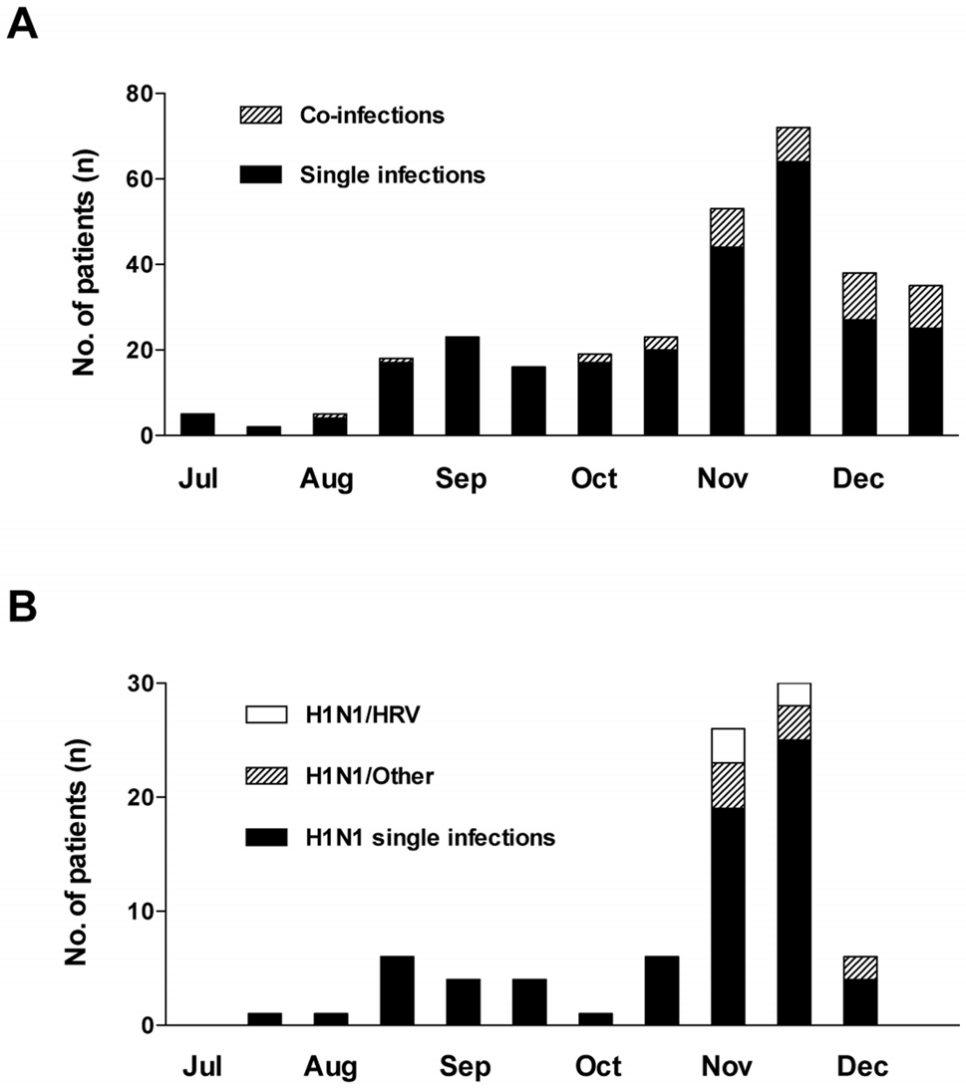
Co-infections over time. Number of children with co-infections over time in (a) all children and (b) H1N1-positive children. H1N1-positive co-infected children divided into co-infection with HRV (H1N1/HRV) and co-infection with other viruses (H1N1/other).

**Table 2:** Distribution of virus in H1N1-positive and H1N1-negative samples.

Detected virus	Totaln	H1N1-negativen (%)	H1N1-positiven (%)	p-value
HAdV	29	26 (11.5)	3 (3.6)	**0.046**
HBoV	29	25 (11.1)	4 (4.8)	0.12
HCoV	17	16 (7.1)	1 (1.2)	**0.049**
HEV	13	13 (5.8)	0 (0.0)	**0.023**
HMPV	2	1 (0.4)	1 (1.2)	0.47
HRV	141	136 (60.2)	5 (6.0)	**<0.0001**
RSV	12	12 (5.3)	0 (0.0)	**0.041**
PIV1	22	21 (9.3)	1 (1.2)	**0.012**
PIV2	9	9 (4.0)	0 (0.0)	0.12
PIV3	2	2 (0.9)	0 (0.0)	1.00
Co-infections		33 (14.6)	12 (14.5)	0.26
Single infections		193 (85.4)	71 (85.5)	
No. of positive samples		226	83	
Total no. of samples		419	83	

Demographic and clinical data were collected from the medical records. Data on vaccination were available for the children with H1N1 but were not systematically collected for all admitted children. Children were categorized according to underlying condition: 1) previously healthy, 2) asthma including children with multiple episodes of obstructive bronchitis, 3) neuromuscular disease, 4) immunosuppression, 5) preterm birth, congenital heart disease and chronic respiratory disease, and 6) other underlying conditions such as renal, metabolic and gastrointestinal diseases. Clinical manifestations during admission were further classified as: 1) uncomplicated (admitted for observation, slight dehydration), 2) respiratory problems such as wheezing, tracheitis, croup and obstructive apnea, 3) suspected bacterial infections such as pneumonia, empyema, bacterial tonsillitis and otitis media, 4) seizures (both febrile and afebrile), 5) other manifestations (gastroenteritis, ileus, metabolic acidosis, myocarditis, renal failure, encephalitis, viral meningitis and myositis). Moreover, admission to the pediatric intensive care unit (PICU) and duration of hospitalization were recorded.

**Figure 3:**
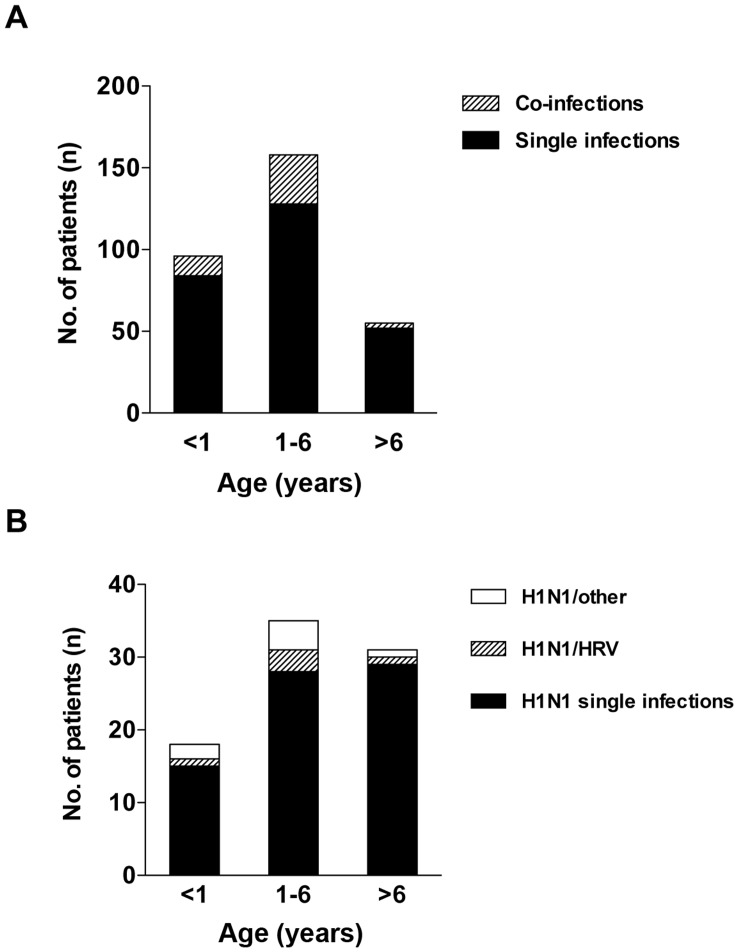
Co-infections and age. Comparison of number of co-infections according to age between children under 1 year (<1), children 1–6 years (1–6) and children over six years (>6) in (a) all children and (b) H1N1-positive children. H1N1-positive co-infected children divided into co-infection with HRV (H1N1/HRV) and co-infection with other viruses (H1N1/other).

**Table 3:** Severity of disease and underlying conditions in children admitted with influenza-like illness.

	H1N1 single infection (n = 71)median/n (%)		H1N1 coinfection (n = 12)median/n (%)	p-value	
Age	3.5	2.7			0.25
Male sex	41 (57.7)	8 (66.7)			0.75
UNDERLYING DISEASE					
Healthy	40 (56.3)	6 (50.0)			0.68
Asthma	11 (15.5)	0 (0.0)			0.35
Neuromuscular disease	6 (8.5)	3 (25.0)			0.12
Immunosuppression	5 (7.0)	1 (8.3)			1.00
Prematurity/VOC/chronic respiratory disease	4 (5.6)	2 (16.7)			0.21
Other^a^	5 (7.0)	0 (0.0)			1.00
SEVERITY OF DISEASE					
Uncomplicated	34 (47.9)	3 (25.0)			0.21
Respiratory problems	14 (19.7)	3 (25.0)			0.70
Bacterial infections	8 (11.3)	4 (33.3)			0.07
Seizures	4 (5.6)	1 (8.3)			0.55
Other^b^	9 (12.7)	1 (8.3)			1.00
Intensive care	7 (9.9)	2 (16.7)			0.61
Days admitted (range)	2 (1–23)	3 (1–12)			0.45
Deaths	2 (2.8)	0 (0.0)			1.00

a Renal, metabolic and gastrointestinal diseases.

b Including gastroenteritis, ileus, metabolic acidosis, myocarditis, renal failure, encephalitis, viral meningitis and myositis.

### Ethics Statement

The study was part of a large retrospective review of clinical data covering 20 years of influenza epidemics in Stockholm approved by the Regional Ethics Review Board in Stockholm (dnr2009/1878-31/1). The guardians consented verbally to the collection of microbiology samples and were informed about the results at the time of the child’s admission according to routine clinical practice. Written consents from the guardian’s were not collected due to the nature of the study.

### Sampling and Microbiological Analyses

Respiratory samples were collected either at the emergency unit or at the pediatric inpatient ward. The majority of samples were obtained from nasopharyngeal aspirates (n = 427) or nasopharyngeal swabs (n = 60) and a few were obtained from tracheal secretion (n = 11), oropharyngeal secretion (n = 2) or bronchoalveolar lavage (n = 2). The samples were extracted with MagAttract Virus Mini M48 kit (Qiagen) in a BioRobot M48 instrument and analyzed by an in-house real-time PCR with a virus panel including the following 16 viruses: influenza A seasonal (FluA) as well as influenza A(H1N1)pdm09 (H1N1), influenza B (FluB), human adenovirus (HAdV), human rhinovirus (HRV), parainfluenzavirus 1–3 (PIV1-3), human bocavirus (HBoV), human metapneumovirus (HMPV), respiratory syncytial virus (RSV), human enterovirus (HEV) and coronavirus serotypes 229E, HKU1, NL63 and OC43 (HCoV) as previously described [15]. The PCR was mainly based on separate single PCRs, however duplex PCR was used for PIV1 and PIV3 and for PIV2 and HCoV-229E. The assay was limited at distinguishing HRV-RNA from HEV-RNA due to cross-reactivity [15]. Detection of both HEV and HRV was determined as only HRV positive if the cycle threshold value (Ct-value) for HRV was at least 5 cycles lower than for HEV. If HEV and HRV were found at equally high reactivity (ΔCt-values <5), the infection was clinically determined as HRV when the predominant symptoms were respiratory tract infection and as only HEV when the predominant symptoms were fever and malaise with clinical suspicion of encephalitis or if HEV was also detected in stool or cerebrospinal fluid. If only a smaller virus panel including H1N1, FluA, FluB and RSV was initially performed, the analyses were complemented for the other viruses. Respiratory samples as well as extracted DNA were stored at −70°C.

### Data Analysis

Data were analyzed in STATA version 12.0 and R version 1.40. Continuous data were analyzed using the Mann-Whitney U-test and categorical variables using Fisher exact test or chi-square test as appropriate. In order to address temporal variations and age co-infections were studied for every half-month and stratification for age (<1 year, 1 to 6 years and >6 years of age) was performed.

## Results

### Viral Findings in 502 Respiratory Samples from Children with ILI

During the study period 559 respiratory samples were analyzed for respiratory viruses. Twenty-three samples tested solely for H1N1 and RSV were excluded due to insufficient material for analysis of additional viruses, 14 samples were taken as surveillance samples without clinical suspicion of influenza and 20 samples were excluded from children with repeated sampling. In total, 502 unique children were included in the analyses, representing a large majority of the 599 admissions with a discharge diagnosis of infection or respiratory disease (ICD-10 diagnosis of A, B or J) during this period. The PCR panel was positive for at least one virus in 309 of the 502 episodes (61.6%). Out of the 16 viruses analyzed, all were detected during the study period except FluA, FluB and HCoV-NL63. HRV was the most frequent pathogen overall (45.6% of the PCR-positive samples, 28.1% of all samples, n = 141) followed by H1N1 (26.9% of the PCR-positive samples, 16.5% of all samples, n = 83) (Table 1). The most frequently detected virus at a specific time point was H1N1 at the peak of the pandemic in November 2009 (Fig. 1). When studying the number of PCR detections over time of HRV and H1N1, the viruses followed a similar pattern coinciding in time with the highest peak in November, also when most children were sampled (Fig. 1).

### Analysis of H1N1/HRV Co-infections and Viral Dynamics

A single virus was detected in 264 samples (85.4% of the PCR-positive samples), two viruses in 41 samples (13.3%), three viruses in 3 (0.9%) and four concurrent viruses in one (0.3%) sample. HEV, RSV, PIV2, H1N1 and HRV were mainly found as single infections (in 92.3%, 91.7%, 88.9%, 85.5% and 78.7% respectively) whereas HBoV and HCoV were more rarely detected alone (in 37.9% and 35.3% of the samples respectively) (Table 1).

The number of co-infections increased through the study period with a significantly higher proportion of co-infections in October-December compared to July-September (p = 0.0008) (Fig. 2a). Co-infections in the H1N1-positive children were exclusively detected in October-December (Fig. 2b). Of the 83 H1N1-positive children 12 (14.5%) were co-infected with another virus of which 5 (6.0%) were positive for HRV, these co-infections all occurred in November, the peak of detection for both viruses (Fig. 2b). When H1N1-negative children where compared to H1N1-positive children, HAdV, HCoV, HEV, HRV, PIV1 and RSV where significantly more often detected in H1N1-negative children (Table 2).

### Viral Findings and Age

Children positive for H1N1 were older (median age 3.3 years) than children positive for HCoV (median age 2.0, p = 0.02), HEV (median age 1.3, p<0.01), HRV (median age 1.5 years, p<0.001), and RSV (median age 0.4, p<0.0001), respectively (Table S1). In an age-stratified analysis, children between 1–6 years (i.e. the usual age of daycare attendance in Sweden) had a significantly higher proportion of co-infections compared to the rest of the children (p = 0.02), however there was no significant age difference when analyses where restricted to the H1N1-positive children (p = 0.34) (Fig. 3).

### Host Factors and Severity of Disease in Relation to Viral Findings

Among the 502 children included in the study 51.4% were reported as previously healthy. The most common underlying condition was asthma or obstructivity (17.9%), followed by neurological disorders (10.9%). Children with asthma or obstructivity had a higher proportion of HRV and HBoV compared to non-asthmatic children (p<0.0001 and p = 0.003 respectively). Among the 83 children with H1N1 infections, 13 (15.7%) had been vaccinated, however, all within less than two weeks prior to admission. Host factors (age, sex and underlying conditions) as well as severity of disease were not found to be associated with co-infections in children with H1N1, except for a tendency of increased frequency of suspected bacterial infections in H1N1-coinfected children (p = 0.07) (Table 3).

Children with H1N1 were treated more often (10.8%) at the pediatric intensive care unit (PICU) than children with other viruses (3.3%) or negative PCR (2.6%) (p = 0.01 and p = 0.004 respectively). The most common PCR finding in the 21 children admitted to PICU during the study period was H1N1 (42.9%), followed by HRV (28.6%). Co-infections were seen in two of the children (9.5%), 14 were single infections (66.7%) and five children were PCR-negative (23.9%). Moreover, nine (42.9%) of the PICU treated children were reported as previously healthy and the most common underlying condition was neurological disorders (23.8%). Six of the children (four with terminal cancer and two with cerebral palsy) died during admission; two with H1N1, one PIV1, one HRV and two with negative PCR.

## Discussion

Among 502 hospitalized children with ILI in Stockholm during the H1N1 pandemic in 2009, 61.6% had a respiratory virus detected by PCR and 16.5% were infected with H1N1. We assessed the impact of co-infections among these children over time and in relation to severity of disease. Although the retrospective study design has limitations with clinical data not being systematically collected, the strength of the study was the uniquely high sampling coverage with extended viral PCR-analyses in admitted children, as a result of the hospital policy during the H1N1 pandemic. The study thus provides a rather complete assessment of the viral panorama in hospitalized children with ILI in Northern Stockholm during this six-month period. Interestingly, among the positive samples only 26.9% were positive for H1N1, suggesting that several other respiratory viruses caused ILI.

Co-infections with two or more viruses were detected in 14.6% of the positive samples, and 14.5% of the H1N1 infected children were positive for an additional virus. Some viruses were more often found in combination with another virus e.g. HBoV and HCoV, while H1N1, HEV, HRV, PIV2 and RSV were mostly detected as single respiratory viral infections. Furthermore, when H1N1-negative children where compared to H1N1-positive children, HAdV, HCoV, HEV, HRV, RSV and PIV1 where more often detected in H1N1-negative children which suggest an association of these viruses with ILI. Although attempts have been made to attribute diseases to individual viruses by quantitative assessments of the PCR results (CT-values) [16] the single time point measurements cannot distinguish which virus that causes the disease. Our results indicate that the causal role of other respiratory viral infections needs to be considered when estimating morbidity attributed to H1N1.

Co-infections might be a result of acquiring two viruses concurrently. Nonetheless, detection of two viruses might rather be a result of a combination of a newly acquired virus together with an asymptomatic infection or shedding from a recent symptomatic infection. HRV is commonly detected in asymptomatic individuals and viral shedding is known to occur for several weeks after an infection [16]–[18], and can be prolonged in asthmatic children [19]. Co-infections were here mainly detected in children 1–6 years of age, indeed the age group attending daycare in Sweden and known to be at high risk of respiratory viral infections. The increasing frequency of co-infections during the study period might also represent accumulation of persistent viral shedding during the autumn.

The children infected with H1N1 were older than children infected with HCoV, HEV, HRV and RSV. H1N1-positive children where also more often treated in the PICU compared to children with other viral infections indicating the pathogenicity of H1N1 in this population. Detection of additional viruses in H1N1-positive children were, as opposed to previous findings [11], [20], not associated with the severity of disease. One explanation could be the low rate of RSV infections in our material since the study period included only the beginning of the RSV season and hence none of the H1N1-positive children were co-infected with RSV. In South America the two epidemics of H1N1 and RSV coincided with a reported high morbidity and a high frequency of severe disease [20]. Unfortunately, bacterial cultures where not systematically performed during the study period. This might bias our results as it has been reported that both influenza and HRV are associated with increased risk for bacterial infections, such as *Streptococcus pneumoniae* [21], [22].

The importance of viral interference for the spread of respiratory disease needs further understanding, as does the role of co-infections in terms of severity of disease. Although a few epidemiological studies have assessed the role of viral interference during the H1N1 pandemic and pointed out HRV as a potential protective factor for H1N1 infection, the results and conclusions drawn have been diverging [7]–[11], [23]. As opposed to previous reports of low prevalence of H1N1 (or even reduction of H1N1) when HRV was prevalent [7]–[9], our data indicated co-circulation of the two viruses and 6.0% of the children with H1N1 were also positive for HRV. Our observation is in line with a recent report from Beijing [24].

The high detection of HRV during the study period might reflect seasonality of HRV disease, but could also be an effect of increased sampling due to the fear of influenza A(H1N1)pdm09 during the pandemic. Indeed, the frequency of HRV-detection seems to closely follow the sampling frequency in children at our hospital throughout the year (unpublished data).

The viruses detected in these hospitalized children are likely to only partly reflect the spread of different viruses in the catchment area during the study period. Our data can therefore not be used to address whether viral interference prevented the spread of H1N1 in Stockholm. It might be tempting to use hospital data to calculate the observed number of co-infections in relation to expected numbers based on the assumption that infections with different viruses occur independently of each other within the total number of hospital specimens [9], [23], [25], [26]. However, these estimates do not take into account the incidence in the catchment population nor the hospitalization rate of the children infected by the respective viruses.

Interestingly, in a recent intervention study, children vaccinated with inactivated trivalent influenza vaccine carried a higher risk of future infections with respiratory picornaviruses compared to children who received placebo [27], suggesting possible interactions between influenza and other viruses. Sweden adopted countrywide vaccination against H1N1 reaching a coverage of approximately 60% of the population, with the first doses distributed in the middle of October 2009 [6] which might have affected the distribution of respiratory viruses in the studied population.

Our data indicate that in addition to H1N1, ILI during the pandemic was associated with a large number of other respiratory viruses. Viral co-infections in children with H1N1 were not associated with severity of disease; moreover the findings of additional viruses in H1N1-positive children need to be taken into account when attributing morbidity to H1N1. To further investigate interaction of respiratory viruses, population-based prospective studies with longitudinal sampling would be highly informative. Moreover the biological mechanism in viral interference needs to be better understood.

## Supporting Information

Table S1 **Age distribution.**
(DOCX)

## References

[1] Guan Y, Vijaykrishna D, Bahl J, Zhu H, Wang J, et al (2010) The emergence of pandemic influenza viruses. Protein Cell 1: 9–13. 21203993 10.1007/s13238-010-0008-z PMC4875113

[2] Dawood FS, Jain S, Finelli L, Shaw MW, Lindstrom, et al (2011) Emergence of a Novel Swine-Origin Influenza A (H1N1) Virus in Humans. N Eng J Med 360: 2605–2615. 10.1056/NEJMoa0903810 19423869

[3] Itoh Y, Shinya K, Kiso M, Watanabe T, Sakoda Y, et al (2009) In vitro and in vivo characterization of new swine-origin H1N1 influenza viruses. Nature 460: 1021–1025. 19672242 10.1038/nature08260 PMC2748827

[4] Falagas ME, Cholevas NV, Kapaskelis AM, Vouloumanou EK, Michalopoulos A, et al (2010) Epidemiological aspects of 2009 H1N1 influenza: the accumulating experience from the Northern Hemisphere. Eur J Microbiol Infect Dis 29: 1327–1347. 10.1007/s10096-010-1002-3 20623384

[5] Swedish Institute for Communicable Disease Control (2010) Influensarapport vecka 1 (4/1–10/1). [article in swedish] http://smi.se/publikationer/veckorapporter/influensarapporter/sasongen20092010/influensarapport-vecka1-2010/. [cited 7 june 2012].

[6] Ortqvist A, Berggren I, Insulander M, de Jong B, Svenungsson B (2011) Effectiveness of an adjuvanted monovalent vaccine against the 2009 pandemic strain of influenza A(H1N1)v, in Stockholm County, Sweden. Clin Infect Dis 52: 1203–1211. 21507917 10.1093/cid/cir182

[7] Linde A, Rotzen-Ostlund M, Zweygberg-Wirgart B, Rubinova S, Brytting M (2009) Does viral interference affect the spread of influenza? Euro Surveill 40:pii = 19354. 19822124

[8] Ånestad G, Nordbø SA (2011) Virus interference. Did rhinoviruses activity hamper the progress of the 2009 influenza A (H1N1) pandemic in Norway? Med Hypotheses 77: 1132–1134. 21975051 10.1016/j.mehy.2011.09.021

[9] Casalegno JS, Ottmann M, Duchamp MB, Escuret V, Billaud G, et al (2010) Rhinoviruses delayed the circulation of the pandemic influenza A (H1N1) 2009 virus in France. Clin Microbiol Infect 16: 326–329. 20121829 10.1111/j.1469-0691.2010.03167.x

[10] Nisii C, Meschi S, Selleri M, Bordi L, Castilletti C, et al (2010) Frequency of detection of upper respiratory tract viruses in patients tested for pandemic H1N1/09 viral infection. J Clin Microbiol 48: 3383–3385. 20592147 10.1128/JCM.01179-10 PMC2937695

[11] Esper FP, Spahlinger T, Zhou L (2011) Rate and influence of respiratory co-infection on pandemic (H1N1) influenza disease. J Infect 63: 260–266. 21546090 10.1016/j.jinf.2011.04.004 PMC3153592

[12] DaPalma T, Doonan BP, Trager NM, Kasman LM (2010) A systematic approach to virus-virus interactions. Virus Res 149: 1–9. 20093154 10.1016/j.virusres.2010.01.002 PMC7172858

[13] Wagner RR (1960) Viral interference, some considerations of basic mechanisms and their potential relationship to host resistance relationship to host resistance. Bacteriol Rev 24: 151–166. 16350163 10.1128/br.24.1.151-166.1960 PMC441044

[14] Statistics Sweden (2009) Population by municipality, marital status, age and sex. http://www.scb.se/[cited 7 june 2012].

[15] Tiveljung-Lindell A, Rotzén-Ostlund M, Gupta S, Ullstrand R, Grillner L, et al (2009) Development and Implementation of a Molecular Diagnostic Platform for Daily Rapid Detection of 15 Respiratory Viruses. J Med Virol 175: 167–175. 10.1002/jmv.21368 PMC7167155 19031448

[16] Jansen RR, Wieringa J, Koekkoek SM, Visser CE, Pajkrt D, et al (2011) Frequent Detection of Respiratory Viruses without Symptoms: Toward Defining Clinically Relevant Cutoff Values. J Clin Microbiol 49: 2631–2636. 21543571 10.1128/JCM.02094-10 PMC3147826

[17] van der Zalm MM, van Ewijk BE, Wilbrink B, Uiterwaal CS, Wolfs TF, et al (2009) Respiratory pathogens in children with and without respiratory symptoms. J Pediatr 154: 396–400. 18823911 10.1016/j.jpeds.2008.08.036 PMC7094528

[18] Wright PF, Deatly AM, Karron RA, Belshe RB, Shi JR, et al (2007) Comparison of results of detection of rhinovirus by PCR and viral culture in human nasal wash specimens from subjects with and without clinical symptoms of respiratory illness. J Clin Microbiol 45: 2126–2129. 17475758 10.1128/JCM.02553-06 PMC1933022

[19] Kling S, Donninger H, Williams Z, Vermeulen J, Weinberg E, et al (2005) Persistence of rhinovirus RNA after asthma exacerbation in children. Clin Exp Allergy 35: 672–678. 15898992 10.1111/j.1365-2222.2005.02244.x

[20] Libster R, Bugna J, Coviello S, Hijano DR, Dunaiewsky M, et al (2010) Pediatric hospitalizations associated with 2009 pandemic influenza A (H1N1) in Argentina. N Eng J Med 362: 45–55. 10.1056/NEJMoa0907673 20032320

[21] McCullers J a, McAuley JL, Browall S, Iverson AR, Boyd KL, et al (2010) Influenza enhances susceptibility to natural acquisition of and disease due to Streptococcus pneumoniae in ferrets. J Infect Dis 202: 1287–1295. 20822454 10.1086/656333 PMC3249639

[22] Wang JH, Kwon HJ, Jang YJ (2009) Rhinovirus enhances various bacterial adhesions to nasal epithelial cells simultaneously. Laryngoscope 119: 1406–1411. 19434681 10.1002/lary.20498

[23] Greer RM, McErlean P, Arden KE, Faux CE, Nitsche A, et al (2009) Do rhinoviruses reduce the probability of viral co-detection during acute respiratory tract infections? J Clin Microbiol 45: 10–15. 10.1016/j.jcv.2009.03.008 PMC7185458 19376742

[24] Yang Y, Wang Z, Ren L, Wang W, Vernet G, et al (2012) Influenza A/H1N1 2009 Pandemic and Respiratory Virus Infections, Beijing, 2009–2010. PloS One 7: e45807. 23029253 10.1371/journal.pone.0045807 PMC3447804

[25] Tanner H, Boxall E, Osman H (2012) Respiratory viral infections during the 2009–2010 winter season in Central England, UK: incidence and patterns of multiple virus co-infections. Eur J Clin Microbiol 31: 3001–3006. 10.1007/s10096-012-1653-3 PMC7088042 22678349

[26] Brunstein JD, Cline CL, McKinney S, Thomas E (2008) Evidence from multiplex molecular assays for complex multipathogen interactions in acute respiratory infections. J Clin Microbiol 46: 97–102. 17977985 10.1128/JCM.01117-07 PMC2224244

[27] Cowling BJ, Fang VJ, Nishiura H, Chan KH, Ng S, et al (2012) Increased risk of noninfluenza respiratory virus infections associated with receipt of inactivated influenza vaccine. Clin Infect Dis 54: 1778–83. 22423139 10.1093/cid/cis307 PMC3404712

