# Correction: Respiratory Viruses in Hospitalized Children with Influenza-Like Illness during the H1n1 2009 Pandemic in Sweden

**DOI:** 10.1371/annotation/fcd5bca6-cbc4-493e-9083-d11903bcbc48

**Published:** 2013-07-15

**Authors:** Samuel Rhedin, Johan Hamrin, Pontus Naucler, Rutger Bennet, Maria Rotzén-Östlund, Anna Färnert, Margareta Eriksson

The name of a virus subtype was given incorrectly in the title. The correct title is: Respiratory Viruses in Hospitalized Children with Influenza-Like Illness during the H1N1 2009 Pandemic in Sweden. The correct citation is: Rhedin S, Hamrin J, Naucler P, Bennet R, Rotzén-Östlund M, et al. (2012) Respiratory Viruses in Hospitalized Children with Influenza-Like Illness during the H1N1 2009 Pandemic in Sweden. PLoS ONE 7(12): e51491. doi:10.1371/journal.pone.0051491. 

