# Supplementary material for: Respiratory Viruses in Hospitalized Children with Influenza-Like Illness during the H1n1 2009 Pandemic in Sweden
Source: PLoS One. 2012 Dec 14;7(12):e51491. doi: 10.1371/journal.pone.0051491 (PMC3522717; doi:10.1371/journal.pone.0051491)
Supplement: Table S1 — Age distribution. (DOCX) [file pone.0051491.s001.docx]

**Table S1.** Age distribution

|  | PIV3  n=2 | RSV  n=12 | HEV  n=13 | HRV  n=141 | HBoV  n=29 | HAdV  n=29 | HCoV  n=17 | PIV1  n=22 | HMPV  n=2 | H1N1  n=83 | PIV2  n=9 |
| --- | --- | --- | --- | --- | --- | --- | --- | --- | --- | --- | --- |
| MEDIAN | 0.2 | 0.4 | 1.3 | 1.5 | 1.7 | 1.9 | 2.0 | 2.2 | 3.2 | 3.3 | 3.7 |
| IQR | 0.1-0.2 | 0.2-1.6 | 0.4-2.7 | 0.8-2.4 | 1.1-3.4 | 1.3-5.8 | 1.2-2.4 | 1.2-3.3 | 3.0-3.5 | 1.3-8.0 | 1.0-9.7 |
